# Effect of supplementation with dried fruit pomace on the performance, egg quality, white blood cells, and lymphatic organs in laying hens

**DOI:** 10.1016/j.psj.2021.101278

**Published:** 2021-05-23

**Authors:** Ewa Sosnówka-Czajka, Iwona Skomorucha

**Affiliations:** Department of Poultry Breeding, National Research Institute of Animal Production, Krakow, Poland

**Keywords:** hen, dried pomace of fruit, lymphatic organ, egg quality

## Abstract

The aim of the current study was to assess the impact of raspberry (**RA**), black currant (**BC**), and black chokeberry (**CA**) dried pomace, at 30 g per kg of feed, on performance, egg quality, white blood cells, heterophil:lymphocyte ratio, and lymphatic organs in Hy-Line laying hens. Hens fed a basal diet comprised the control group (**CO**). A total of 480, 42-week-old Hy-Line commercial hybrid laying hens were randomly divided into 4 equal groups differing in the diet: in control group (**CO**), the birds received a standard diet, in groups CH, BC and RA, the birds were fed with a standard diet enriched with 30 g/kg of dried fruit pomace: Black chokeberry, black currant, and raspberry, respectively.

Differences in yolk color were observed between the CO and groups BC and RA, as well as between groups CA and BC (*P* < 0.001). The eggs of group RA were characterized by a lower weight (*P* = 0.001 and *P* = 0.002) and shell density (*P* = 0.025 and *P* = 0.002) in comparison to eggs from the control group CO. The lightest yolk color was observed in the eggs of group BC in comparison with the other groups (*P* = 0.006). The greatest yolk weight was observed for group CH, while the smallest was for group CO (*P* = 0.017). Laying hens in the groups BC and RA were characterized at wk 62 of age by a higher percentage of spleen in comparison with the control group CO (*P* = 0.018).

In conclusion, the BC and RA diet resulted in a paler yolk color and was associated with slightly poorer shell quality parameters. On the other hand, the CH and BC diet had a positive impact on the immune system of the laying hens, as confirmed by the changes in the white blood cell smear and the higher spleen percentage. Therefore, the use of these by-products in the diets of poultry, i.e., dried pomace of black chokeberry and black currant may have a positive impact by improving the immunological status of laying hens.

## INTRODUCTION

The fruit processing industry generates more than 0.5 billion tons of waste worldwide, and the global availability of this material and its unused potential have encouraged researchers to conduct numerous studies on the possible utilization of this waste (Banerjee et al., 2017). “Zero waste” is a trend that is growing increasingly popular with consumers and advocates a lifestyle that minimizes the production of waste. As defined by the Zero Waste organization, zero waste means the conservation of all resources by means of responsible production, consumption, reuse, and recovery of products, packaging, and materials (ZWIA, 2018). This trend also entails the reuse of fruit pomace, including reuse in the food industry (Tańska et al., 2016), or in biorefineries and in the production of biogas (Banerjee et al., 2017).

Poland, due to its location in a temperate climate zone, is a significant producer of chokeberries, raspberries, and black currants. The majority of these crops are processed, leading to large quantities of by-product such as pomace which still retain valuable properties as a source of many substances, including dietary fiber, minerals, antioxidants, and vitamins. Fruit and vegetables are rich in bioactive compounds that can help prevent many degenerative diseases (Shashirekha et al., 2015). These compounds are also present, often at even higher concentrations, in the by-products of fruit and vegetable processing (Sójka et al., 2013). One way of fruit industry by-products utilization is as feed additives in poultry rations. These additives may have a positive impact on the metabolism and health of animals and on product quality (Juskiewicz et al., 2017).

Pomaces of black chokeberry, raspberry, and black currant contain a very high proportion of seeds. As is suggested by the literature, seeds obtained as by-products of the processing of fruit, mainly berry fruits, are a valuable source of oils with a unique composition of fatty acids occurring in conjunction with high concentrations of fat-soluble antioxidants, primarily tocopherols (Pieszka et al., 2015; Mildner-Szkudlarz et al., 2019).

Black chokeberry (*Aronia melanocarpa*) is one of the richest plant sources of dietary fiber, sorbitol, and phenol phytochemicals (procyanidins, anthocyanins, proanthocyanidins, phenolic acids and flavonols) with numerous healing and therapeutic effects, e.g., antioxidant (Bednarska and Janiszewska-Turak, 2020; Dąbrowski et al., 2020), anti-inflammatory (Zapolska-Downar et al., 2012; Iwashima et al., 2019), anticarcinogenic, cardioprotective, antimutagenic, hepatoprotective, and antidiabetic effects (Kedzierska et al., 2012; Worsztynowicz et al., 2014; Iwashima et al., 2019; Wenzel et al., 2020). Chokeberry is not consumed in its fresh form but rather after processing, mainly in the form of a juice which may be used in processed products in order to increase their antioxidant properties (Banach et al., 2020; Sidor et al., 2020). However, significant amounts of these important nutrients and bioactive substances are not squeezed into the chokeberry juice, but instead remain in the by-products of juice processing (D'Alessandro et al., 2014). Kitrytė et al. (2017) subjected chokeberry pomace to an extraction process in order to obtain bioactive substance. These extracts contained mainly phenolic acids and flavonols. The authors also stated that all fractions of the chokeberry pomace could be used as an inexpensive source of functional components with high added value.

The limited number of studies dealing with the use of raspberry, black currant, and chokeberry pomace in poultry feeds inclined the authors to conduct a study to assess the impact of the dietary addition of raspberry, black currant, and black chokeberry dried pomace on performance and selected parameters of egg quality, white blood cells, heterophil: Lymphocyte ratio and lymphatic organs in laying hens.

## MATERIALS AND METHODS

### Experimental Site

Experimental studies were conducted at the Experimental Poultry Farm of the National Research Institute of Animal Production in Aleksandrowice, Poland.

### Animals, Diets and Experimental Design

The experimental protocol was approved by the II Local Ethics Committee for Animal Experiments in Kraków (Resolution no. 422). All study procedures and animal care activities were performed in accordance with the guidelines for the care and use of experimental animals.

Experimental material comprised 480, 42-week-old Hy-Line commercial hybrid laying hens. The birds were placed in a 3-tier enriched cage battery of enhanced cages in which there was 750 cm^2^ of surface area for each hen. Birds were randomly divided into 4 equal groups differing in the diet: in group I, the control group (**CO**), the birds received a standard diet for laying hens (Table 1), in groups II (**CH**), III (**BC**) and IV (**RA**), the birds were fed with a standard diet enriched with 30 g/kg of dried fruit pomace (Tables 2 and 3), respectively: Black chokeberry (CH), black currant (BC), and raspberry (RA) respectively. Each kilogram of standard feed was mixed with 30 g of dried fruit pomace at the experimental feed production plant of the National Research Institute of Animal Production in Aleksandrowice, and later the feed was added to the troughs in experimental groups. The hens received feed with the addition of dried fruit pomace during the period from 42 to 62 wk of age.

**Table 1:** Composition and analysis of the standard diet fed to laying hens.

Item	Content
Ingredients (g/kg)	
Maize	370.0
Wheat	225.0
Soybean meal (46% CP)	240.0
Alfalfa	20.0
Rapeseed oil	28.0
Ground limestone	88.0
Dicalcium phosphate	1.90
Sodium chloride	3.0
DL-Methionine	1.4
Vitamin-mineral premix*	5.0
Nutritive value per kg⁎⁎	
Metabolizable energy (MJ)	11.60
Crude protein (g)	175.0
Lysine (g)	8.60
Methionine (g)	4.10
Calcium (g)	37.00
Phosphorus (g)	4.00

⁎ Provided per kilogram of diet: vit. A 10000 IU; vit. D_3_ 2500 IU; vit. E 25 IU; Mn (manganese oxide) 85 mg; Mn (dimanganese trihydroxychloride) 15 mg; Cu 15 mg; Fe 70 mg; Zn 80 mg; I 1.5 mg; Se 0.2 mg; Ca min. 0.84 g; Na 1.57 g; crude protein 1.45 g; crude fiber 0.002 g; ash 6.60 g; crude fat 0.072 g; lysine 0.3 g; methionine 1.5 g; ME 19.08 kCal.

⁎⁎ Nutritive value per kg was calculated.

**Table 2:** Analysis of the experimental diet fed to laying hens.

Nutritive value per kg	Diet of dried pomace of black chokeberry	Diet of dried pomace of black currant	Diet of dried pomace of raspberry
Metabolizable energy (MJ)	12.10	12.21	12.32
Crude protein (g)	177.9	179.7	178.1
Calcium (g)	37.05	37.06	37.07
Phosphorus (g)	4.06	4.11	4.06

**Table 3:** Nutritive value, fatty acid profile and level of anthocyanins in the experimental dried fruit pomace.

Item	Dried pomace of black chokeberry	Dried pomace of black currant	Dried pomace of raspberry
Nutritive value per kg			
Dry matter (g)	929.8	938.9	939.3
Crude protein (g)	96.3	158.1	102.9
Crude fat (g)	51.6	70.5	115.3
Crude fiber (g)	199.2	267.5	465.0
Calorific value (cal/g)	4858.00	4847.00	5746.12
Calcium (mg/g)	1.74	1.95	2.30
Magnesium (mg/g)	-	1.24	1.08
Phosphorus (mg/g)	1.92	3.77	1.83
Fatty acid profile (% of total fatty acids)			
C 16	5.48	12.11	4.19
C 16-1 *(n-7)*	0.19	0.36	0.14
C 18	1.71	1.66	1.19
C 18-1 *(n-9)*	16.38	10.47	11.70
C 18-2 *(n-6)*	43.43	41.56	49.01
gamma18-3*(n-6)*	0.03	8.01	0.04
C 18-3 *(n-3)*	29.78	15.17	33.02
C 18-4 *(n-3)*	0.02	2.14	0.00
C 18:2 (CLA) c9-c11	0.01	0.00	0.00
C 20	1.52	2.37	0.45
C 20-1	0.33	0.76	0.00
C 20-2 *(n-6)*	0.14	0.38	0.00
C 20-4 *(n-6)*	0.00	0.00	0.00
C 22	0.59	2.55	0.13
C 22-5 *(n-3)*	0.00	0.00	0.09
C 22-6 (DHA) *(n-3)*	0.00	0.01	0.01
SFA	9.51	19.33	5.95
UFA	80.67	90.49	94.05
PUFA	69.06	73.57	82.17
PUFA n-6	49.94	43.60	49.05
PUFA n-3	19.12	29.97	33.12
PUFA n-6/n-3	2.61	1.45	1.48
UFA/SFA	8.48	4.68	15.81
PUFA/SFA	7.26	3.81	13.81
Sum of anthocyanins (mg/100 g of product)	768.36	205.20	3.16

Each group was comprised of 12 cages (area: 112 × 67 cm, height: 45 cm) and in each cage (replicate) there were 10 birds (480 hens in total). Cages were equipped with nipple drinkers, a feed trough, nest, perch, claw trimmer, and scratching mat. The birds had free access to feed and water. The lighting program for laying hens during the experimental period was 16 h of light and 8 h of darkness per day, while temperature and humidity were roughly 16°C and 65%.

### Chemical Analyses

The samples of dried fruit pomace were analyzed for basic composition, level of minerals (calcium, magnesium, potassium), fatty acid profile, and anthocyan content (Table 2). All the analyses were performed in accordance with the methods described by Pieszka et al. (2015).

Basic composition of dried pomaces was determined by standard methods (AOAC, 1995). Mineral content was determined by atomic absorption spectrometry (**AAS**) on ICP-MS spectrophotometer after previous mineralization in a microwave oven (Milestone Ethos Plus, Sorisole, Italy). The concentration of anthocyans was determined using a JASCO V-530 spectrophotometer (Jasco, Tokyo, Japan). The fatty acid profile of the pomace samples was determined using gas chromatography (Varian 3400, Woonsoket, RI).

### Sample Collection and Laboratory Analyses

During the experiment, production results were monitored. Feed intake, egg production from each replicate was monitored daily. Eggs were collected daily and weighed individually (Vibra SJP-6200 CE). Feed conversion efficiency (g/egg and kg/kg of eggs) was calculated by measuring the feed consumption and number of eggs and egg weights.

At wk 55 and 62, eggs were collected from each cage (replicate). Next, 20 eggs were randomly selected from each group and evaluated according to the method reported by Sokołowicz et al. (2018) for the following parameters: weight (g), shape index (%), yolk and shell percentage in the whole egg; eggshell traits: color intensity (%), weight (g), thickness (μm), density (mg/cm^2^), breaking strength (N); physical features of egg content: albumen height (mm), value of Haugh units (**HU**), yolk color (scores according to a 15-point DSM scale). Egg weight was determined by weighing individually with a digital laboratory balance exact to 0.1 g. Shape index of eggs was determined as a ratio of short-to-long axis which was measured using an electronic caliper (MITUTOYO Absolute Digmatic Caliper model CD-15DCX, Kawasaki, Japan) exact to 0.01 mm. Percentage contents of egg morphological components (albumen, yolk and shell) were calculated based on their weights measured individually for each egg. Eggshell color, weight, density and thickness, HU value, yolk color according to DSM scale were measured using electronic equipment for egg quality measurements (EQM – Egg Quality Measurements, Technical Services and Supplies, Crawley, UK). Eggshell strength (N) was measured using a multipurpose testing system, model BT1-FR1.OTH.D14 with measuring head 100N and software testXpert (Zwick/Roell GmbH&Co.KG, Ulm, Germany).

In addition, 1 bird (10 birds/group) was randomly selected at wk 55 and 62 from 10 cages (replicates) and individually weighed (WPT/F-30C). Birds were stunned with an STZ 6 electric stunner and their blood was collected by decapitation into tubes containing EDTA as an anticoagulant. The collected blood was used to perform manual blood smears using two glass slides, including one with cut edges. The smears were prepared and dried before staining with May-Grünwald-Giemsa (Alpha Diagnostics Warszawa, Poland, Cat. No LO 001, LO 002). The stained leukocytes (heterophils H, lymphocytes L, monocytes, basophils, and eosinophils) were counted under a Nikon YS 100 microscope using the Schilling differential method (differential white blood cell count). The cells were counted using hematology counter (SH 12/12, Poland). The heterophil:lymphocyte (**H:L**) ratio was calculated by dividing the number of heterophils by the number of lymphocytes.

From the 10 birds decapitated for blood collection, the lymphatic organs (spleen, bursa of Fabricius) and all thymus lobes were dissected. Each organ was stripped of adhering tissue and then weighed individually with a precision balance (WPS-360/C/2) and their percentage of total body weight was calculated as:

$$\%\;lymphatic\;organ\;=\frac{lymphatic\;organ\;weight\;\left(g\right)}{live\;weight\;before\;slaugtering\;\left(g\right)}\cdot \;100$$

### Statistical Analysis

Prior to testing, the Shapiro-Wilk test was used to assess the normality of distribution. The test confirmed that the data analyzed conformed to a normal distribution. Raw data were analyzed for outliers (mean ± 2.5 SD). Significant outliers were not included in the mean results and statistical analysis. Based on the results obtained, for each of the properties mentioned above, the following were calculated: mean values and the pooled standard error of the mean (Pooled SEM), which were tabulated and presented in the subsection Results. The experimental units were tissues collected from birds (blood sample, lymphatic organ), egg, as well as a cage with 10 birds for the data on laying performance, feed intake and feed conversion. The results were statistically analyzed by one-way ANOVA with diets as fixed effects. Significant differences for the means between the experimental groups were determined with Duncan's Multiple Range Test. The *P* < 0.05 was considered as a significant difference, and a value between *P* > 0.05 and *P* < 0.10 was considered as a trend toward significance. Statistical analyses were carried out using the Statistica software version 12.0 PL (StatSoft Inc., 2011, Tulsa, OK).

## RESULTS

No differences were observed in the production results (*P* ˃ 0.05) of laying hens in the period between the 42 and 62 wk of age (Table 4). However, the weight of eggs from groups CH and BC tended to be higher than in the control group CO (*P* = 0.064). In Table 5 the quality of the eggs collected at 55 and 62 wk is presented. At wk 55 of the experiment, differences were observed in the egg shape index (1.92%) between CH and BC (*P* = 0.012). Differences were also noted in the yolk color between the control group CO and the groups fed with BC (1.45 DSM) and RA diet (1.15 DSM) with a *P* value of *P* < 0.001, as well as between the group fed with CH and BC diet (0.85 DSM) with a *P* value of *P* < 0.001. The eggs collected from group RA were also characterized at this time by a lower weight and lower shell density in comparison to the control group CO (*P* = 0.001 and *P* = 0.025 respectively). At wk 62 of the experiment, differences in shell color were noted between the eggs of the group fed with CH and RA diet (*P* = 0.009). The palest yolk color was observed in the eggs of group BC in comparison with the other groups (*P* = 0.006). The highest yolk weight was observed for eggs in the group fed CH diet, while the the lowest for the CO group (*P* = 0.017). The eggs collected from the laying hens in the group fed with RA and BC diet were characterized by a lower shell weight compared to the eggs of the CO group (*P* = 0.002). A lower shell density was noted in relation to the CO group for eggs from the hens fed with RA diet (*P* = 0.002). Shell percentage was found to be lower for the eggs from hens fed diets BC and RA compared to the CO group (*P* = 0.022). A 0.59% difference was observed in the percentage of shell weight in egg weight between groups CH and BC (*P* = 0.022). No differences were found between the groups for the other quality parameters (*P* ˃ 0.05)

**Table 4:** Effect of diet supplemented with 30 g/kg of dried fruit pomace on performance of laying hens.

	Group^1^					
Item	CO	CH	BC	RA	Pooled SEM	*P*-value
Egg production (%)^2^	89.1	84.8	86.9	85.5	1.57	0.758
Feed intake (g) per bird/d^2^	115.40	116.52	115.61	118.73	1.85	0.998
Feed conversion (g) per egg^2^	129.56	138.62	133.16	134.91	2.82	0.779
Feed conversion (kg) per kg of eggs^2^	2.04	2.08	1.99	2.06	0.06	0.939
Egg weight (g)^3^	65.84	66.33	66.88	65.44	0.68	0.064

1 CO, control group - the birds’ feeds were a standard feed mixture for laying hens; CH, the birds’ feeds were supplemented with 30 g/kg of dried pomace of black chokeberry; BC, the birds’ feeds were supplemented with 30 g/kg of dried pomace of black currant; RA, the birds’ feeds were supplemented with 30 g/kg dried pomace of raspberry.

2 n = 12 per group.

3 n = 120 per group. Abbreviation: SEM, the pooled standard error of the mean.

**Table 5:** Effect of diet supplemented with 30 g/kg of dried fruit pomace on quality of eggs (n = 20 per group).

		Group^1^					
Item	Week of rearing	CO	CH	BC	RA	Pooled SEM	*P*-value
Shape index (%)	55	78.10 ^a,b^	77.75^a^	79.67^b^	78.71^a,b^	0.58	0.012
Shell strength (N)		40.57	43.77	44.45	40.26	1.75	0.546
Shell color (%)		29.75	29.65	29.00	31.55	1.07	0.379
Egg weight (g)		65.14	63.83	64.25	62.81	1.02	0.443
Albumen height (mm)		7.27	7.39	7.98	7.60	0.34	0.473
Haugh units (HU)		82.58	84.03	87.19	85.70	2.00	0.416
Yolk color (DSM)		10.10^b^	9.50^b,c^	8.65^a^	8.95^a,c^	0.22	<0.001
Yolk weight (g)		17.56	17.34	17.29	16.99	0.31	0.642
Yolk (%)^2^		27.02	27.20	26.99	27.10	0.22	0.986
Shell thickness (µm)		385.60	381.05	381.40	371.30	4.95	0.222
Shell weight (g)		7.51^a^	7.18^a,b^	7.24^a,b^	6.98^b^	0.14	0.001
Shell (%)^2^		11.53	11.26	11.29	11.15	0.09	0.438
Shell density (mg/cm^2^)		94.25^a^	91.62^a,b^	90.46^a,b^	88.50^b^	1.66	0.025
Shape index (%)	62	77.75	77.10	77.80	76.95	0.62	0.754
Shell strength (N)		32.20	36.05	34.30	37.35	2.38	0.497
Shell color (%)		27.95^a,b^	25.65^a^	28.20^a,b^	30.00^b^	1.08	0.009
Egg weight (g)		65.85	67.80	66.00	68.05	1.12	0.368
Albumen height (mm)		6.95	7.35	7.35	7.25	0.33	0.840
Haugh units (HU)		80.65	82.60	82.90	81.25	2.01	0.866
Yolk color (DSM)		10.05^b^	10.05^b^	9.05^a^	10.00^b^	0.23	0.006
Yolk weight (g)		18.10^a^	19.10^b^	18.20^a,b^	18.35^a,b^	0.36	0.017
Yolk (%)^2^		27.02	28.04	26.87	27.13	0.27	0.433
Shell thickness (µm)		375.55	370.65	370.15	369.15	6.12	0.884
Shell weight (g)		7.60^a^	7.30^a,b^	7.10^b^	6.95^b^	0.16	0.002
Shell (%)^2^		11.12^a^	11.00^a,c^	10.41^b^	10.52^b,c^	0.10	0.022
Shell density (mg/cm^2^)		91.35^a^	88.60^a,b^	87.10^a,b^	83.65^b^	1.83	0.002

1 CO, control group - the birds’ feeds were a standard feed mixture for laying hens; CH, the birds’ feeds were supplemented with 30 g/kg of dried pomace of black chokeberry; BC, the birds’ feeds were supplemented with 30 g/kg of dried pomace of black currant; RA, the birds’ feeds were supplemented with 30 g/kg dried pomace of raspberry.

2 Relative weight of yolk and shell were calculated as a percentage of egg weight. Abbreviation: SEM, the pooled standard error of the mean. ^a,b,c^ Means within a row with different superscripts differ (*P* < 0.05).

Table 6 presents the effect of the diet supplemented with 30 g/kg of dried fruit pomace on white blood cells and heterophil:lymphocyte ratios of laying hens. At wk 55, a difference was noted in the percentage heterophil (*P* = 0.004) and monocyte (*P* = 0.003) content in the blood of hens from the control group CO and the group CH. A difference was also noted between these groups in the percentage content of lymphocytes (6.3%), basophils (2.16%), and eosinophils (1.77%) at wk 62 (*P* = 0.022, *P* = 0.026 and *P* = 0.011, respectively). Additionally, a higher percentage of heterophils and eosinophils was noted for hens in the CO group in comparison respectively with hens from BC (*P* = 0.049) and RA groups (*P* = 0.011), respectively.

**Table 6:** Effect of diet supplemented with 30 g/kg of dried fruit pomace on white blood cells (%) and heterophil:lymphocyte (H:L) ratios of laying hens (n=10 per group).

		Group^1^					
Item	Week of rearing	CO	CH	BC	RA	Pooled SEM	*P*-value
Lymphocytes	55	59.89	59.20	60.19	60.67	2.32	0.318
Heterophils		31.16^b^	25.13^a^	28.41^a,b^	29.26^a,b^	1.46	0.004
Monocytes		2.73^a^	4.64^b^	2.90^a^	3.71^a,b^	0.48	0.003
Basophils		2.64	4.16	3.84	3.27	0.69	0.437
Eosinophils		3.11	3.01	3.94	3.09	0.62	0.696
H:L		0.53	0.40	1.06	0.49	0.14	0.359
Lymphocytes	62	56.60^a^	62.90^b^	60.50^a,b^	60.82^a,b^	1.75	0.022
Heterophils		33.12^a^	30.16^a,b^	28.37^b^	30.14^a,b^	1.63	0.049
Monocytes		2.49^a,b^	1.82^a^	3.40^b^	2.57^a,b^	0.47	0.042
Basophils		4.46^a^	2.30^b^	3.92^a,b^	3.59^a,b^	0.59	0.026
Eosinophils		4.57^a^	2.80^b^	3.80^a,b^	3.00^b^	0.47	0.011
H:L		0.56	0.50	0.47	0.52	0.02	0.437

1 CO, control group - the birds’ feeds were a standard feed mixture for laying hens; CH, the birds’ feeds were supplemented with 30 g/kg of dried pomace of black chokeberry; BC, the birds’ feeds were supplemented with 30 g/kg of dried pomace of black currant; RA, the birds’ feeds were supplemented with 30 g/kg dried pomace of raspberry. Abbreviations: H:L, heterophils to lymphocytes ratio; SEM, the pooled standard error of the mean. ^a,b^Means within a row with different superscripts differ (*P* < 0.05).

The effect of the diet supplemented with 30 g/kg of dried fruit pomace on relative weight of lymphatic organs is presented in Table 7. At wk 55 of the experiment, no differences were observed between the groups in the percentage of lymphatic organs (*P* ˃ 0.05). The birds fed with BC and RA diets were characterized at wk 62 by a higher spleen percentage in comparison to the CO group (*P* = 0.018).

**Table 7:** Effect of diet supplemented with 30 g/kg of dried fruit pomace on relative weight of lymphatic organs (%) (n = 10 per group).

		Group^1^					
Item^2^	Week of rearing	CO	CH	BC	RA	Pooled SEM	*P*-value
Spleen	55	0.1005	0.0913	0.0769	0.0825	0.010	0.442
Bursa of Fabricius		0.0114	0.0083	0.0101	0.0085	0.001	0.545
Thymus		0.0647	0.0708	0.0609	0.0727	0.010	0.947
Spleen	62	0.0887^a^	0.1237^b^	0.1200^b^	0.0974^a,b^	0.010	0.018
Bursa of Fabricius		0.0357	0.0104	0.0115	0.02926	0.013	0.531
Thymus		0.0674	0.0865	0.0686	0.0856	0.007	0.194

1 CO, control group - the birds’ feeds were a standard feed mixture for laying hens; CH, the birds’ feeds were supplemented with 30 g/kg of dried pomace of black chokeberry; BC, the birds’ feeds were supplemented with 30 g/kg of dried pomace of black currant; RA, the birds’ feeds were supplemented with 30 g/kg dried pomace of raspberry

2 The lymphatic organs were calculated as percentage of total body weight. Abbreviation: SEM, the pooled standard error of the mean. ^a,b^Means within a row with different superscripts differ (*P* < 0.05).

## DISCUSSION

Kara et al. (2016) showed that supplementation of the diets of laying hens with grape marc at 4% and 6% did not significantly impact the production of eggs or feed consumption. Similarly, Kara and Kocaoğlu-Güçlü (2012) did not note any effect of a 2% grape marc supplement on laying performance, feed conversion, or egg weight. In turn, Ghaemi et al. (2014) did not observe any impact of the diet supplemented with 5 or 10% apple pomace on the production results of laying hens, although a 15% supplement lowered egg weight laying performance, as well as having a detrimental effect on feed conversion. Loetscher et al. (2013) did not observe any impact of the diet supplemented with chokeberry pomace at 25 g/kg on the production results of broilers. Furthermore, Loetscher et al. (2014) did not note effects on laying performance, feed intake or feed conversion per egg in the case of hens receiving a feed with a 2.5% chokeberry pomace. In the present study, differences were also not observed in production results between experimental birds fed with CH, BC, RA diet and the birds of the control group. There was only a tendency for higher egg weight in hens fed the standard diet supplemented with dried black chokeberry and black currant pomace. The black chokeberry and black currant pomace fed to the layers was rich in anthocyans and may have had a positive effect on the overall metabolism, which translated into a tendency for higher egg weight in hens from these experimental groups. Also other authors, who used anthocyan-rich supplements, showed their positive effects on overall poultry performance (Ahossi et al., 2016; Reis et al., 2019).

In the present study, diet with CH generally did not impact on egg quality, in concurrence with the research of Loetscher et al. (2014), who stated that supplementation of the diet of laying hens with a 2.5% chokeberry pomace did not modify egg weight, shell quality, or yolk color. In the present study we only observed that the CH diet had an effect on higher yolk weight at wk 62 of the experiment compared to the eggs from the control group. This is not supported by Loetscher et al. (2014), probably because of the lower (2.5%) proportion of chokeberry pomace in the experimental diet. Also other authors failed to observe any effect of fruit supplements in hen diets on egg quality parameters (Kara and Kocaoğlu-Güçlü, 2012; Tarasewicz et al., 2013; Kara et al., 2016). The fruits of the chokeberry, black currant, and raspberry are rich sources of natural colorants such as carotenoids and anthocyanins (Pieszka et al., 2015; Wilczyński et al., 2017) which should influence the intensity of the yolk color. Kazimierska et al. (2011) state that the intensity of color does not, however, depend only on the level of colorants in the diet, but also on their relative proportions and the ability of the laying hens to to process these. In the present study, a paler yolk color was observed in eggs from hens fed with dried black currant and raspberry pomace, which is not a favorable change from the point of view of the Polish consumer, who generally prefers eggs with a more intensive yolk color (Kazimierska et al., 2011). Moreover, Goliomytis et al. (2018) noted a lesser intensity of yolk color when supplementing fruit pulp to the diets of laying hens.

A significant factor affecting both producers and consumers of eggs is shell quality, i.e., shell weight, thickness, and density. Bozkurt et al. (2012) found that the diet with essential oils from 6 different herbs for laying hens increased the weight, thickness, and strength of the shells. Lewko and Gornowicz (2015) did not observe any impact of the use of herbs or green fodder in the diets of hens on the weight, color, or density of the shells, yet they did observe a significant effect of these additives on an increase in shell thickness. Other authors have also failed to demonstrate the impact of supplementation of hens’ diets on shell quality (Duru, 2013; Tarasewicz et al., 2013; Loetscher et al., 2014; Kara et al., 2016). In turn, in the present study, diet with 30 g/kg dried raspberry pomace decreased the weight and density of the egg shells, which may subsequently result in more breakages during egg collection and sorting.

Klasing (2007) states that a particular diet may have an impact on the immune systems of poultry. The nutrients which exhibit a strong effect on immunostimulation include long-chain polyunsaturated fatty acids (PUFA), carotenoids, flavonoids, vitamins A, C, D and E, and also antioxidants (Klasing, 2007; Ahmed et al., 2014; Islam et al., 2017; Ebrahimzadeh et al., 2018), which typically occur in herbs and fruit. Ahmed et al. (2014) observed an improvement in the immunological status of broiler chickens fed with the citrus fruit yuzu (*Citrus junos)* at 10 and 20 g/kg. Islam et al. (2017) demonstrated that an extract of cranberry may modulate birds’ humoral response to pathogenic viruses. Moreover, Ebrahimzadeh et al. (2018) claim that the diet with grape marc of chickens may increase the cellular and humoral immunity. Similarly, Iqbal et al. (2015) stated that broiler chickens fed a diet enriched with grape polyphenols exhibited higher antibody titers against the ND virus than birds fed a control diet. Additionally, Pourhossein et al. (2015) observed a higher titer of antibodies against all viral strains applied and higher IgG and IgM levels when supplementing the feed with an extract from the skin of the sweet orange (*Citrus sinensis*) at 1250 ppm/kg to feed.

Voslarova et al. (2013) state that one measure of immunological function may be the number of leukocytes present. Islam et al. (2017) demonstrated that a high content of phenols, including anthocyans, enhances the immune status of birds. In the present study, the level of anthocyans was higher for dried black chokeberry and black current pomace compared to dried raspberry pomace. This was reflected in the lower number of heterophils in layers’ blood at wk 55 and a higher number of lymphocytes at wk 62 for diet CH, as well as in the lower number of heterophils in the blood of 62-week-old hens receiving the BC diet compared to the control group. However, this had no effect on the H:L ratio between the groups. Although the differences in the number of heterophils and lymphocytes did not have a clear effect on reducing the H:L ratio in these groups, it can be considered that diets CH and BC had a beneficial effect on the immunity of the experimental laying hens.

The organs of the immunological system are believed to be important sites for the recognition of antigens and the activation of immune cells (Akter et al., 2006), and the relative weight of the spleen and bursa of Fabricius reflect the system's ability to generate T and B lymphocytes during immune response (Ebrahimzadeh et al., 2018). Rahimi et al. (2011) did not observe differences in the relative weight of the spleen and bursa of Fabricius of broilers fed with a feed containing concentrates of grape and thyme in comparison with chickens from the control group. Similar results were obtained by Ebrahimzadeh et al. (2018), who did not observe effects on the relative weight of these organs among birds fed a diet supplemented with grape marc. In the present study, diet with 30 g/kg dried black chokeberry (CH) and black currant (BC) pomace increased the percentage of the spleen in total body weight of birds fed thus in comparison to the control group. However, Al-Harthi (2017) did not observe an impact of diet with the olive cake on the bursa of Fabricius or the spleen of broiler chickens. Moreover, Pourhossein et al. (2015) did not observe effects on the relative weight of the spleen or bursa of Fabricius in broiler chickens fed with an extract of sweet orange.

In summary, the diet of laying hens with 30 g/kg dried fruit pomace from black chokeberry, black currant, and raspberry had generally no effect on the production results. A diet supplemented with dried black currant and raspberry pomace did, however, result in a paler yolk color at wk 55, while at wk 62 a paler yolk color was observed only in the group supplemented with dried black currant pomace. The raspberry pomace diet was also associated with slightly poorer egg quality parameters (shell weight and density) at wk 55 and 62, and also for dried black currant pomace lower shell quality (shell weight and percentage) was observed at wk 62. In turn, dried black chokeberry contributed to higher yolk weight at wk 62. On the other hand, the diet with dried black chokeberry and black currant pomace did have a positive impact on the immune system of the laying hens, as confirmed by the changes in white blood cell smear and by the higher spleen percentage.
